# An improved genome editing system for Sphingomonadaceae

**DOI:** 10.1099/acmi.0.000755.v3

**Published:** 2024-05-13

**Authors:** Inmaculada García-Romero, Rubén de Dios, Francisca Reyes-Ramírez

**Affiliations:** 1 Departamento de Biología Molecular e Ingeniería Bioquímica, Centro Andaluz de Biología del Desarrollo, Universidad Pablo de Olavide/Consejo Superior de Investigaciones Científicas/Junta de Andalucía, 41013 Sevilla, Spain; 2 Division of Biosciences, Department of Life Sciences, Centre of Inflammation Research and Translational Medicine, College of Health, Medicine and Life Sciences,, Brunel University London, Uxbridge, UK

**Keywords:** bioremediation, genetic tools, genome editing, mutation, Sphingomonadaceae, sphingomonads

## Abstract

The sphingomonads encompass a diverse group of bacteria within the family *Sphingomonadaceae*, with the presence of sphingolipids on their cell surface instead of lipopolysaccharide as their main common feature. They are particularly interesting for bioremediation purposes due to their ability to degrade or metabolise a variety of recalcitrant organic pollutants. However, research and development on their full bioremediation potential has been hampered because of the limited number of tools available to investigate and modify their genome. Here, we present a markerless genome editing method for *Sphingopyxis granuli* TFA, which can be further optimised for other sphingomonads. This procedure is based on a double recombination triggered by a DNA double-strand break in the chromosome. The strength of this protocol lies in forcing the second recombination rather than favouring it by pressing a counterselection marker, thus avoiding laborious restreaking or passaging screenings. Additionally, we introduce a modification with respect to the original protocol to increase the efficiency of the screening after the first recombination event. We show this procedure step by step and compare our modified method with respect to the original one by deleting *ecfG2*, the master regulator of the general stress response in *S. granuli* TFA. This adds to the genetic tool repertoire that can be applied to sphingomonads and stands as an efficient option for fast genome editing of this bacterial group.

## Data Summary

All data and protocols used or generated through this work have been provided within this article or in the associated supplementary files.

## Introduction

Sphingomonads are a bacterial group encompassing the genera *Sphingobium*, *Sphingopyxis*, *Novosphingobium*, and *Sphingomonas*, classified within the family *Sphingomonadaceae* [1,2]. The members of the family *Sphingomonadaceae* are Gram-negative alphaproteobacteria of various sizes that do not form spores. They can be motile or non-motile, and when they are, they often have a polar flagellum. The colonies they form exhibit yellow or orange tones due to the presence of carotenoids and contain sphingolipids (glycosphingolipids) in their cell envelopes instead of lipopolysaccharides (reviewed in [3]). Sphingomonads are widely distributed in nature, as they inhabit multiple terrestrial and aquatic environments, and have been isolated from plant roots, clinical samples, and other sources [4]. Some of them have been also found to be endophytic [5,6] and assist in phytoremediation processes (reviewed in [7]), and a facultative anaerobe, *Sphingopyxis granuli* TFA, has been described, which is able to grow in anaerobic conditions in the presence of nitrate [8].

Among the family *Sphingomonadaceae*, sphingomonads are well known for their ability to degrade recalcitrant compounds, including aromatic hydrocarbons. Examples of sphingomonads with this ability are *S. granuli*, which is capable of using tetralin as the sole carbon and energy source [9]; *Sphingomonas aromaticivorans* F199, which degrades biphenyl, naphthalene, m-xylene and p-cresol [10]; *Sphingomonas wittichii* RW1, which catabolises dibenzo-p-dioxin [11]; or *Sphingobium chlorophenolicum* L-1, which catabolises pentachlorophenol [12]. Some members of this group are even able to metabolise pharmaceutical agents, such as *Sphingomonas* sp. MPO218, which utilises ibuprofen as carbon source [13]. Additionally, *Sphingopyxis macrogoltabida* NBRC 15033 and *Sphingopyxis* sp. PVA3 are able to degrade synthetic polymers such as polyethylene glycol (PEG) [14] and polyvinyl alcohol (PVA) [15], respectively, employed in the production of plastic items, adhesives or packaging films [16]. Due to their ability to degrade xenobiotic compounds, this group of bacteria has attracted special interest in the bioremediation field. However, as non-model microorganisms, they have been lagging behind in the development of efficient genetic tools and genome editing technologies. The further development of these tools is essential for fully understanding the biodegradation pathways and physiology of this group of bacteria and to maximise their bioremediation potential.

In this regard, basic genetic tools have been developed for mutational analyses and targeted mutagenesis in *Sphongomonadaceae*, including gene disruption and replacement strategies. However, these methods may cause polar effects on the genes located downstream in an operon. This hindrance led to the development of markerless gene deletion strategies based on a double recombination involving successive selection–counterselection rounds. In a first round, a plasmid harbouring the upstream and downstream homologous regions of the target gene is transferred to the strain of interest and the first recombination into the chromosome is selected, typically using an antibiotic resistance marker. In addition to this selection marker, plasmids used for markerless gene deletion procedures include a counterselection marker that may confer sensitivity to specific compounds, depending on the marker. By growing the cointegrate strain in the presence of this compound, only cells undergoing a second recombination and losing the plasmid backbone will be able to grow. A classic example of a counterselection marker is *sacB*, which confers sensitivity to sucrose and has been applied to different members of the *Sphingomonadaceae* [17,23]. The *sacB* marker confers sensitivity to sucrose, thus growing the cointegrate clones in the presence of this disaccharide after a first recombination would favour a second recombination. This second recombination event would lead either to the reconstitution of the wild-type genomic configuration or to the stable introduction of the aimed genome modification. However, the highly frequent emergence of spontaneous sucrose-resistant mutants and the need of multiple rounds of sub-culturing in the presence of sucrose make this system tedious and poorly reproducible [24]. Kaczmarczyk *et al*. [24] developed a similar strategy with better reproducibility, taking advantage of the natural streptomycin resistance (Str^R^) of sphingomonads, which has been used extensively since its publication [25,40]. In this case, they engineered an artificial allele of *rpsL*, termed *rpsL1*, that confers sensitivity to streptomycin (Str^S^) and can be used as a counterselection marker. Despite the unquestionable technical improvement tested for a range of sphingomonads, the Str sensitivity produced by *rpsL1* varied across species, which may again need further passaging or restreaking to achieve the second recombination.

To accelerate the double-recombination procedure, Martínez-García and de Lorenzo [41] developed a method for genome editing in *Pseudomonas putida*, subsequently modified by Wirth *et al*. [42], in which the second recombination is triggered by a DNA double-strand break. This addition would avoid the need for a counterselection marker, bypassing the restreaking/passaging steps and hence shortening the protocol. In this method, the upstream and downstream homologous regions of the target gene are cloned in a non-replicative plasmid flanked by two SceI restriction sites termed pEMG. After the introduction of this vector, the first recombination event, a second vector carrying the SceI coding gene under an inducible promoter, termed pSW-I, is transferred into the cointegrate strain. The expression of *sceI* produces a double-strand break in the cognate restriction sites that is eventually repaired by homologous recombination, resolving the cointegrate and either producing the deletion of the target gene or a reversal to the wild-type genotype. The strength of this method lies in the need to forcefully repair the double-strand break by recombination, regardless of a counterselection, for which the strain of interest may develop secondary adaptations or resistance mutations. Thanks to its high efficiency and versatility, this genome editing strategy has been extensively applied for single-gene deletions, as well as for the removal of whole-gene clusters and insertion of epitopes in various species [43,48]. Furthermore, it has even been adapted to other bacteria that have remained recalcitrant to gene manipulation, including multidrug-resistant *Acinetobacter baumannii*, in which it has been used to edit the chromosome as well as native plasmids [49].

In this work, we describe the optimisation of the SceI-based genome editing method to *S. granuli* TFA, supporting its further applicability to the family *Sphingomonadaceae*. Furthermore, we implement this strategy with additions from Kaczmarczyk *et al*. to improve the detection of single-recombinant clones. The improvement of this method with respect to traditionally used counterselection-based procedures lies in forcing a second recombination rather than just favouring it. We do this by deleting *ecfG2*, a well-known regulator of the alphaproteobacterial general stress response (GSR) and showing the effect of the deletion on the GSR activation with respect to the wild-type TFA. We also provide guidelines for this procedure in a step-by-step comprehensive protocol.

## Protocol optimization: methods and results

### Plasmid construction

The original plasmids to perform the SceI-based genome editing strategy, pEMG [kanamycin resistance (Km^R^)] and pSW-I [ampicillin resistance (Ap^R^)], were kindly provided by Professor V. de Lorenzo (CNB, Madrid). Plasmids pMPO1409 (Km^R^, carrying upstream and downstream homologous regions to delete *ecfG2* in *S. granuli* TFA), pMPO1408 (Ap^R^, carrying a *ecfG2::lacZ* gene fusion), and pMPO1412 [Km^R^Str^S^ (streptomycin sensitivity)] were previously constructed as described by de Dios *et al*. [50] and González-Flores *et al*. [51], respectively. For the purpose of comparing the original protocol (using pEMG-derivative plasmids) and the subsequent improvements using pMPO1412-derivative plasmids (introducing the *rpsL1* counterselection marker in pEMG), we constructed pMPO1162, a pMPO1412-derivative carrying the above-mentioned *ecfG2* homologous regions. This construction was performed by digesting pMPO1409 with SacI and XbaI (New England Biolabs), purifying a 2 kb fragment containing the *ecfG2* homologous regions using the GFX (GE Healthcare Life Sciences) DNA purification kit, and ligating it into pMPO1412 digested with the same enzymes using T4 DNA ligase (New England Biolabs). Enzymatic reactions and purification procedures were performed as per the manufacturer’s instructions. Chemically competent *Escherichia coli* DH5α λ*pir* were transformed with ligation mixtures via heat shock transformation.

### Use of *rpsL1* counterselection improves the screening of cointegrate candidates

**Fig. 1.**
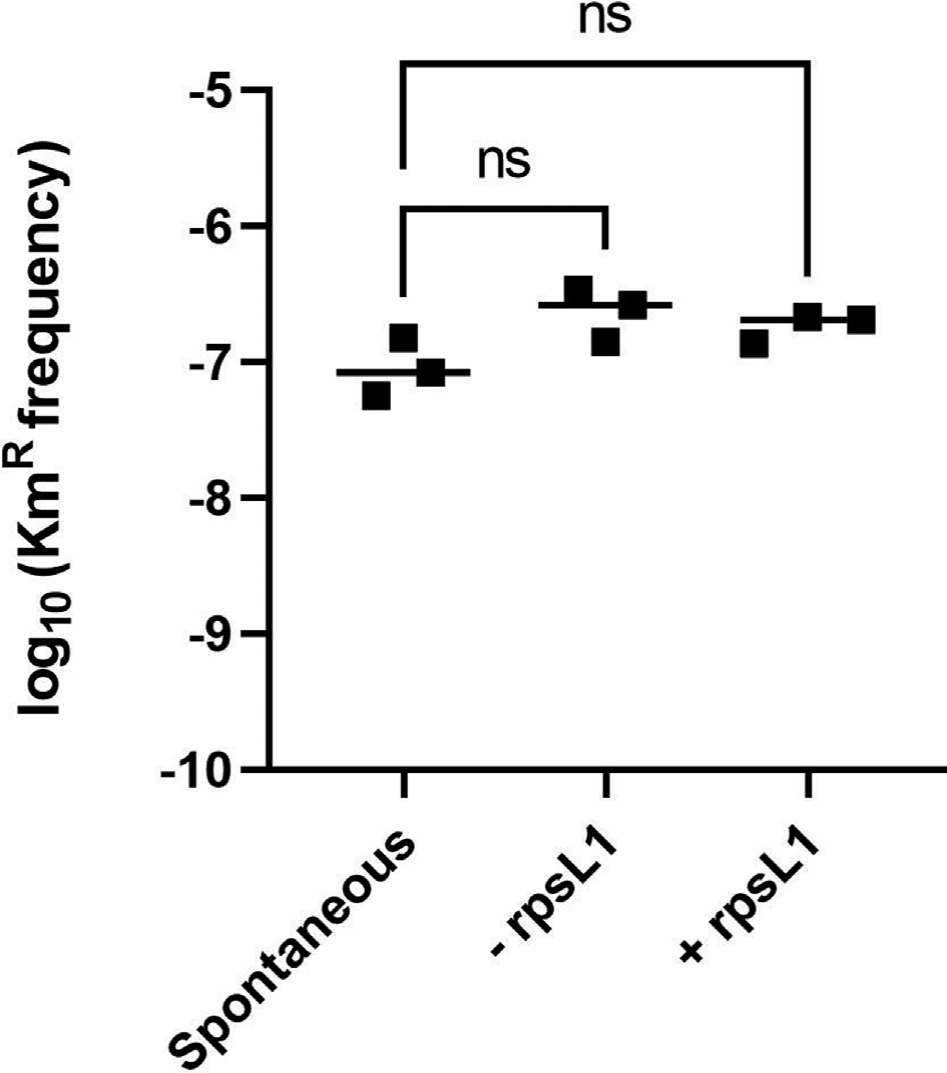
Logarithmic representation of *S. granuli* transformation frequency when transforming with pMPO1409 (*−rpsL1*) and pMPO1162 (*+rpsL1*) compared to a control (spontaneous). Two hundred nanograms of the respective plasmid (an equivalent volume of water in the case of the control) were used in each case. Each transformation was repeated with three independently prepared aliquots of electrocompetent *S. granuli* TFA cells. Individualised colony counts for each repeat and frequency calculations are shown in Table S1, available in the online version of this article. One-way ANOVA was performed between the different samples. ns, non-significant.

Traditionally, Km has been used as a selection marker in sphingomonads in general and in *S. granuli* in particular. However, when selecting single-recombination events, the recombination frequency is similar to that of spontaneous kanamycin-resistant mutants (Fig. 1). Due to this, the distinction of cointegrates carrying pEMG derivatives with the upstream and downstream regions of the target gene required tedious screening by PCR. In our efforts to make this step more efficient, we cloned the *rpsL1* counterselection marker in pEMG, obtaining pMPO1412 in previous work [51]. In order to compare both approaches, we attempted to delete *ecfG2* using backbone plasmids with and without carrying the *rpsL1* allele in parallel. To do this, *S. granuli* TFA was electrotransformed with 200 ng of pMPO1409 or pMPO1162, or an equivalent amount of bi-distilled water as a control. All transformations were performed in biological triplicate and serial dilutions were plated on MML agar supplemented with 20 mg l^−1^ Km (plain MML agar for viable cell counting). As a result, we confirmed that the spontaneous emergence of Km^R^ mutants in the control transformations was not significantly different from that obtained in the transformations with pMPO1409 and pMPO1162.

**Fig. 2.**
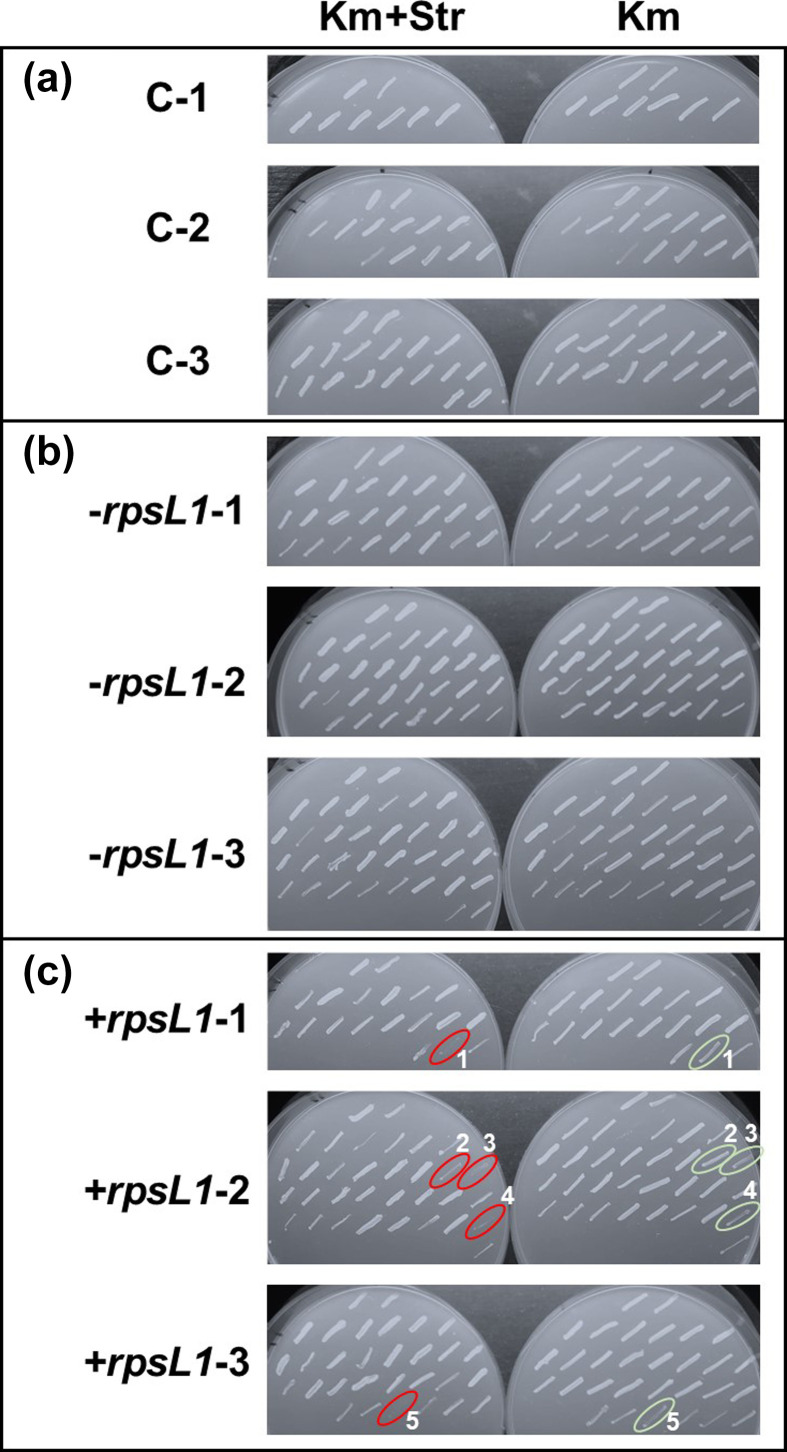
Streaks of clones obtained from the control electroporation (**a**), or electrotransformations with pMPO1409 (*−rpsL1*) (**b**) or pMPO1162 (+*rpsL1*) (**c**). The red-labelled streaks do not grow or grow slowly with Km 20 mg l^−1^ plus Str 200 mg l^−1^ when compared to the same streaks growing with only Km 20 mg l^−1^ (labelled in green). Results for the previously mentioned three independent electrotransformation replicates are shown, as well as controls in which the electroporation was performed with an equivalent volume of water.

To identify the cointegrate clones, we performed a screening with all the resulting kanamycin-resistant colonies by streaking them on MML agar plates supplemented with either 20 mg l^−1^ Km alone or with 20 mg l^−1^ Km and 200 mg l^−1^ Str (four times the concentration of Str we routinely use to select the wild-type TFA strain), which would negatively impact the growth of the clones carrying the *rpsL1* marker. Then, the agar plates were incubated at 30 °C for just 16 h (all the streaks would look equally grown if incubated beyond 18–20 h). As expected, colonies taken from control plates and those transformed with pMPO1409 grew at similar rates in media supplemented with Km only or with Km and Str (Fig. 2a, b). However, plates streaked with colonies obtained by transforming TFA with pMPO1162, harbouring *rpsL1*, showed multiple clones that grew visibly more slowly in the presence of Km and Str compared to those grown in the presence of Km only (Fig. 2c).

**Fig. 3.**
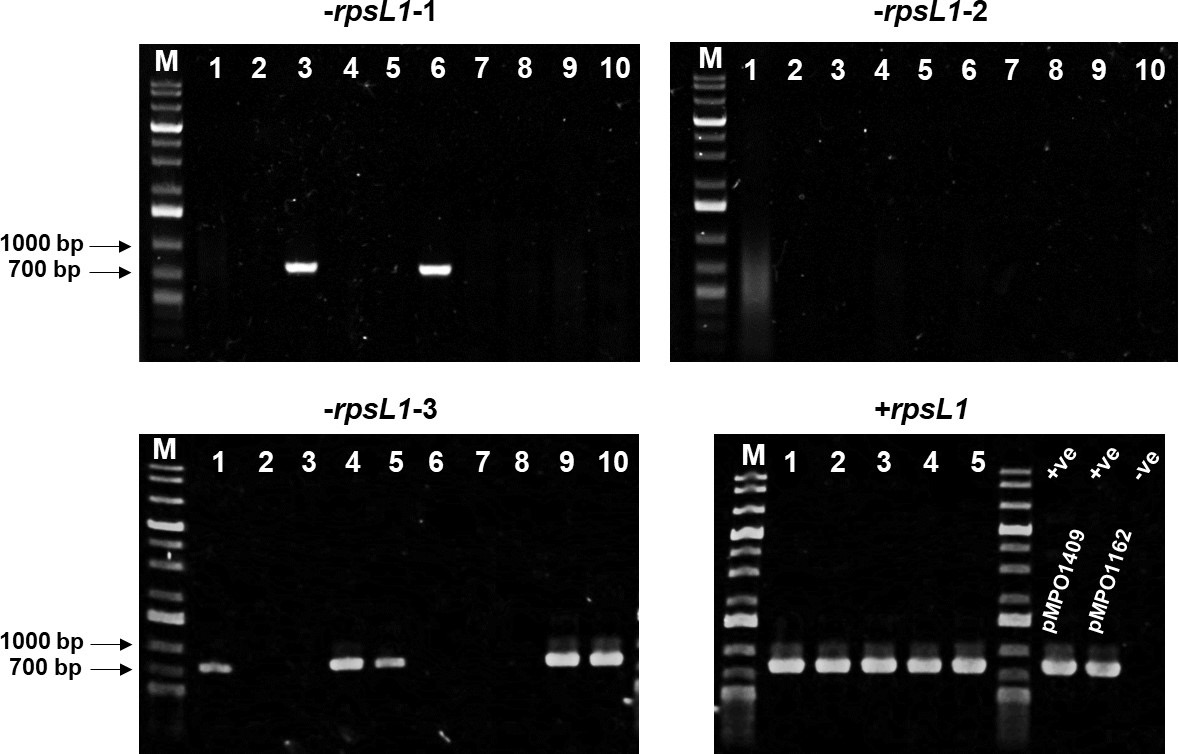
Agarose gel electrophoresis (0.8 %) of PCR products from different clones after electrotransformation with pMPO1409 (*−rpsL1*) or pMPO1162 (+*rpsL1*), using the primers KmFw (GATTGAACAAGATGGATTGC) and KmRev (CGTCAAGAAGGCGATAGAAGG). The plasmids pMPO1409 and pMPO1162 were used as positive (+ve) controls, respectively, and no DNA was added in the negative (−ve) control.

To test the efficacy of this counterselection as an improvement to the screening process, we performed an additional PCR screening using primers that annealed within the Km^R^ marker harboured in pMPO1409 and pMPO1162, yielding an amplicon of approximately 700 bp (KmFw: GATTGAACAAGATGGATTGC; KmRev: CGTCAAGAAGGCGATAGAAGG). To do this screening, we randomly selected 10 clones from each of the 3 pools obtained by transformation with pMPO1409, as well as 5 targeted clones transformed with pMPO1162 that grew more slowly in the presence of Km and Str. As shown in Fig. 3, only 7 out of 30 clones transformed with pMPO1409 yielded a PCR product, indicative of having undergone the first recombination event. However, all five clones tested from the transformation with pMPO1162 yielded a PCR product. This conclusively shows how using the *rpsL1* counterselection as an indication of the first recombination event reduces the number of clones to test and yields more targeted and efficient screening.

### Transformation with pSW-I triggers the second recombination

**Fig. 4.**
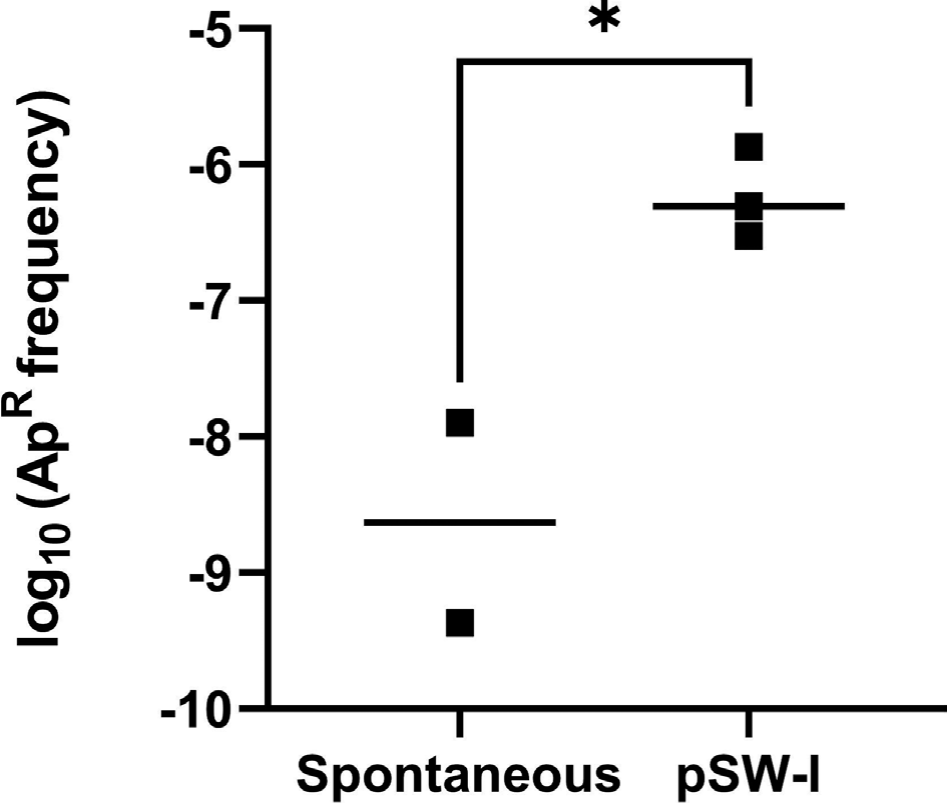
Logarithmic representation of transformation frequency when introducing pSW-I into each independent cointegrate clone compared to the respective negative controls. Two hundred nanograms of plasmid were used in each case, and an equivalent amount of water was used for the negative controls. Individualised colony counts for each repeat and frequency calculations are shown in Table S2. Student *t*-test analysis was performed between the different samples. *, *P*<0.05.

At this point, the plasmid carrying the upstream and downstream homologous regions of the target gene (*ecfG2* in this case) flanked by the SceI restriction sites are inserted into the TFA chromosome by a single recombination event. The next step consists of forcing the second recombination that leads to the *ecfG2* deletion. To do this, a replicative plasmid carrying the *sceI* gene needs to be transferred into the selected cointegrate clone. To perform this step, we selected clones 3, 4, and 5 as labelled in Fig. 2c, prepared electrocompetent cells of each of them, and transformed them with 200 ng of pSW-I in parallel to the respective three controls with an equivalent amount of water. Selection was carried out on MML agar supplemented with Ap 5 mg l^−1^. In this case, the transformation frequency with pSW-I was significantly higher than the emergence of spontaneous ampicillin-resistant clones (Fig. 4).

In the original *P. putida* protocol, both the pEMG derivative construct and the pSW-I plasmid are simultaneously selected prior to inducing the *sceI* expression. However, during our first attempts to use this procedure, we observed very poor growth when selecting both genetic elements at the same time. A possible explanation would be that the presence of both the cointegrate and pSW-I simultaneously has a strong fitness cost due to the leaky expression of *sceI*. This would continuously produce double-strand breaks in the SceI target site introduced in the chromosome with the first recombination step. For this reason, rather than selecting the presence of both elements at the same time as in the original protocol [41], we selected only the presence of pSW-I. In addition to this, the original protocol requires the induction of the *sceI* expression by adding 3-methylbenzoate. This was further optimised by Wirth *et al*. [42] by doing the pSW-I selection and *sceI* induction in a single step. However, during the optimisation of this protocol for use on *S. granuli* TFA, we noticed that the leaky expression of *sceI* alone was ufficient to trigger the DNA double-strand break. This has also been observed for other bacteria, for which the addition of 3-methylbenzotae is even deleterious for growth [49].

For these reasons, we directly screened the Ap^R^, Km^S^ clones obtained after the transformation with pSW-I (50 clones per transformation) by streaking them on MML agar plates supplemented with either 20 mg l^−1^ Km, 5 mg l^−1^ Ap or 50 mg l^−1^ Str (Fig. S1). After this, we observed that, for each of the three independent pSW-I transformations, 48, 62, and 30 % of the clones were Km^S^, indicating that they had undergone a second recombination event during the selection process.

**Fig. 5.**
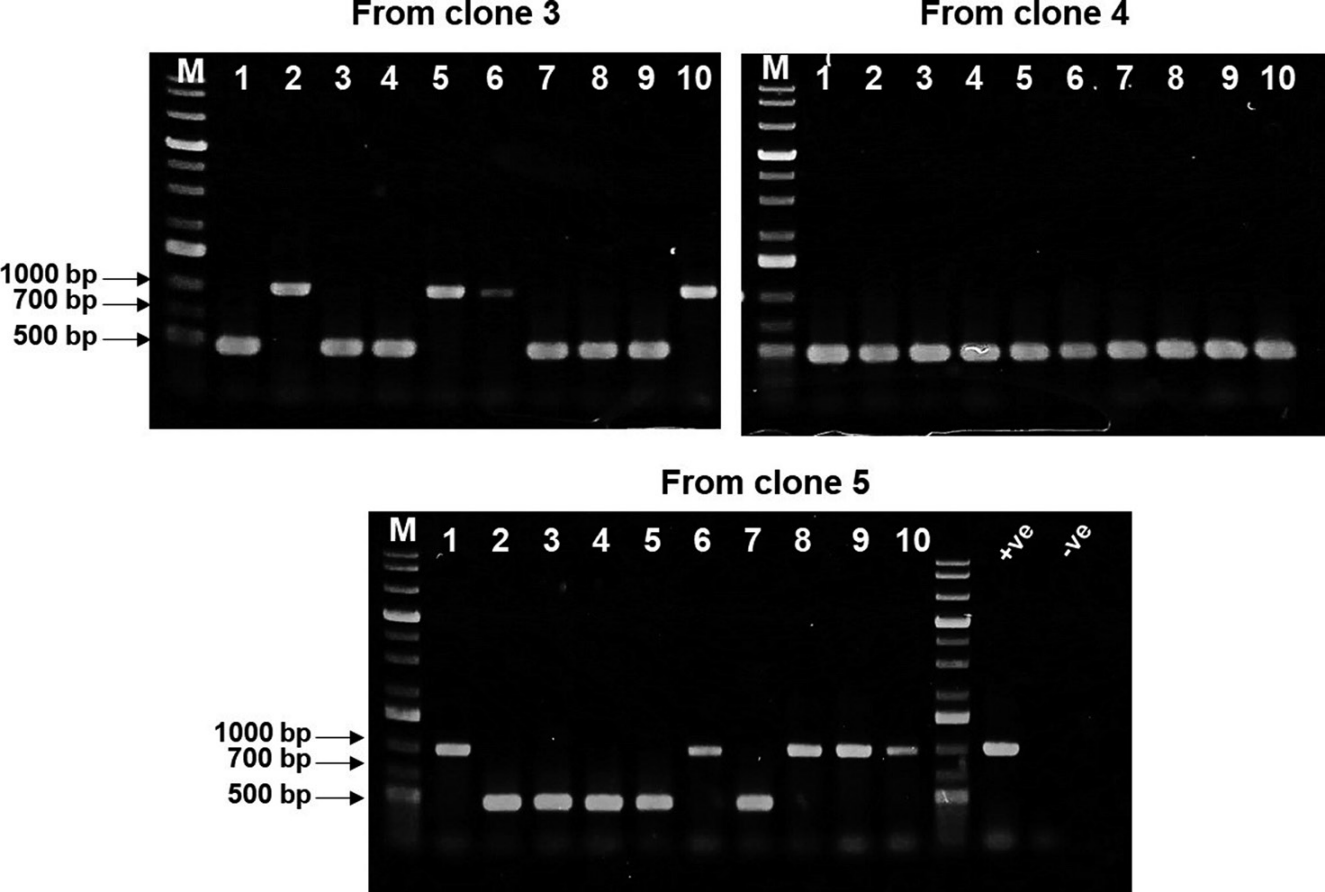
Agarose gel electrophoresis (0.8 %) of PCR product from different clones of second recombination event, using the oligos Seq_ecfG2_Fw2 (ACCGATTTTGCCCATGGCTTC) and Seq_ecfG2_Rv (CGAACGGAAACAGAGGTGATC). The plasmid used in the electroporation was pSW-I. Genomic DNA from the wild-type TFA strain was used as positive (+ve) control and no DNA was added in negative (−ve) control. The wild-type fragment is ~1000 bp and the deletion of *ecfG2* is shown as a ~500 bp fragment.

To assess whether the second recombination had led to the deletion of *ecfG2* or if the clone had reverted to the wild-type genotype, we screened 10 clones of each transformation by PCR. We used primers Seq_ecfG2_Fw2 (ACCGATTTTGCCCATGGCTTC) and Seq_ecfG2_Rv (CGAACGGAAACAGAGGTGATC), which would yield a product of approximately 1 kb in the case of the wild-type configuration or approximately 0.5 kb in the case of the *ecfG2* deletion. We found that 21 out of 30 total clones had suffered the deletion of *ecfG2* (Fig. 5).

**Fig. 6.**
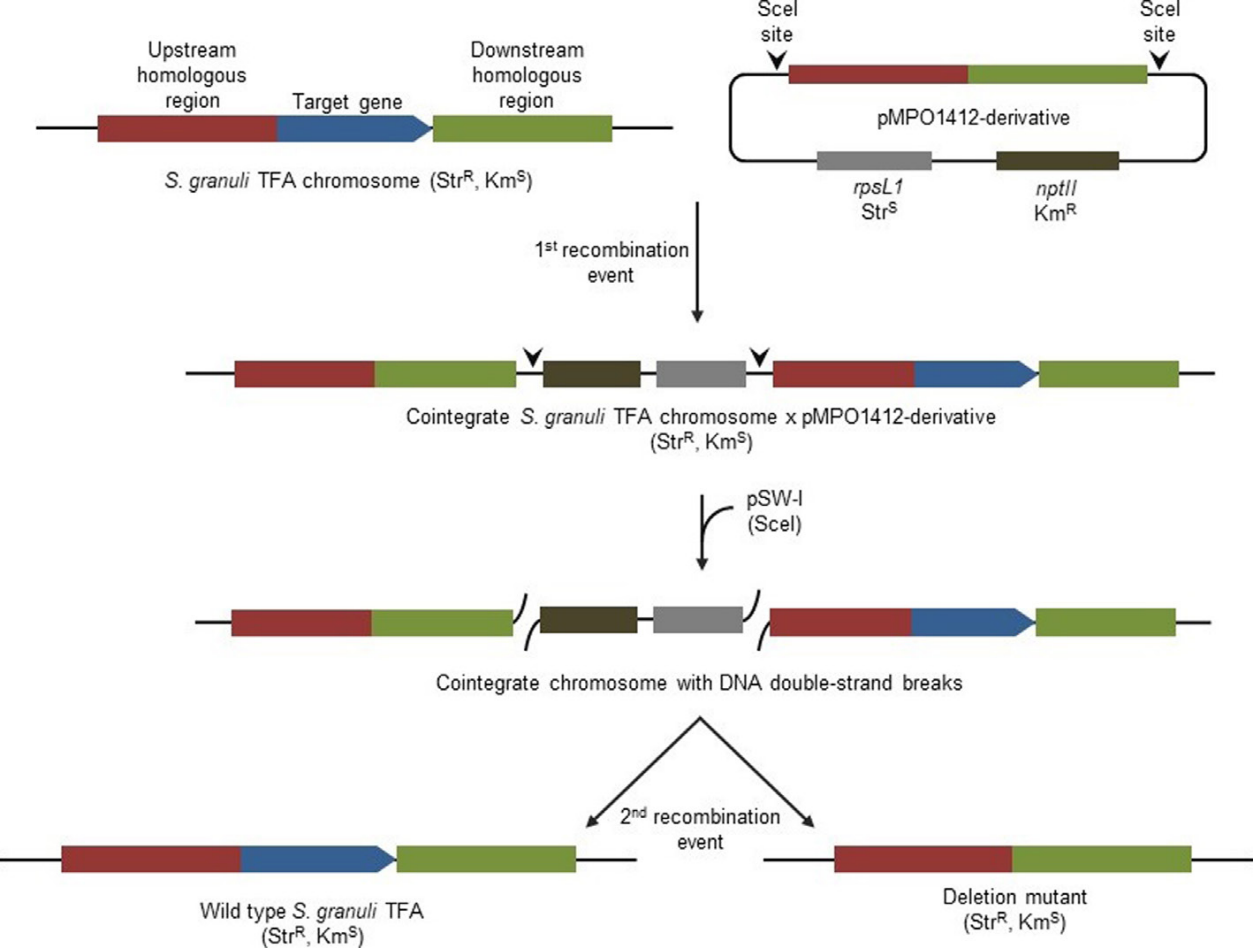
Schematic representation of the genetic rearrangements and recombinations occurring during this genome editing procedure for a generic target gene (*ecfG2* in the example developed in this work), as well as the two possible outcomes: a reconstitution of the wild-type genotype or the aimed genome modification.

As a final step, pSW-I has to be cured from the deletion mutant. To do this, we inoculated one positive clone from each pSW-I transformation in MML broth in the absence of Ap. After two passages, cells were serially diluted and plated on MML agar supplemented with 50 mg l^−1^ Str. To conclusively assess the loss of both pMPO1162 and pSW-I, 50 colonies obtained by plating each individual clone were streaked on MML agar supplemented with either 20 mg l^−1^ Km, 5 mg l^−1^ Ap or 50 mg l^−1^ Str. We found that none of the clones grew in the presence of Km or Ap, but all grew in the presence of Str (Fig. S2), which indicated the curing of all genetic devices used in this deletion strategy. A scheme summarising the recombinations and genetic rearrangements undergone during this procedure is shown in Fig. 6.

### Deletion of *ecfG2* abolishes the expression of *nepR2*

**Fig. 7.**
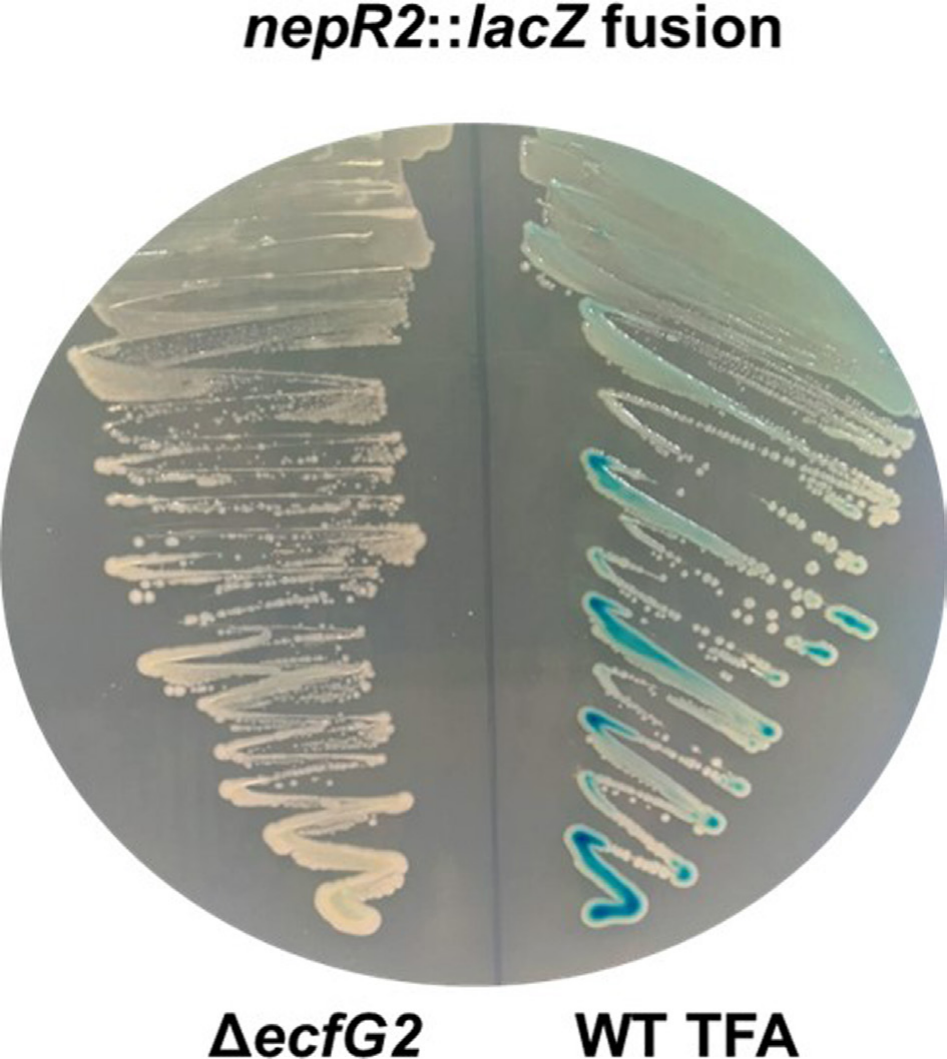
Expression of *nepR2::lacZ* fusion in the mutant Δ*ecfG2* compared to the wild-type (WT) TFA strain. EcfG2 is essential for activating the expression of *nepR2* [50]. Thus, the *nepR2::lacZ* fusion (born in pMPO1408) in the Δ*ecfG2* background does not yield a blue colour in the presence of X-gal. Both strains were streaked on MML agar plates supplemented with X-gal 25 mg l^−1^ and were incubated at 30 °C for 5 days.

EcfG2 is an extracytoplasmic function sigma factor that acts as the main regulator of the GSR in *S. granuli* TFA [49,50]. Although *ecfG2* is essential under stress conditions, a mutant in this gene shows a similar fitness to the wild-type TFA in the absence of stress. Among its target genes, *nepR2* encodes an anti-sigma factor that exerts a negative feedback loop on the activation of the GSR [49]. To show the phenotype of the new constructed Δ*ecfG2* mutant, we transformed the Δ*ecfG2* mutant along with the wild-type TFA with pMPO1408, an integrative vector carrying a *nepR2::lacZ* gene fusion. Thus, EcfG2-mediated activation of the GSR, and thus *nepR2*, would lead to the production of the β-galactosidase enzyme that breaks down X-gal (5-bromo-4-chloro-3-indolyl-β-d-galactopyranoside) into a blue precipitate. To visualize this, we streaked both the wild-type TFA and the Δ*ecfG2* deletion mutant, both carrying the *nepR2::lacZ* fusion, on a MML agar plate supplemented with 25 mg l^−1^ X-gal and incubated it at 30 °C for 5 days. As a result, we observed that the wild-type TFA yielded a blue colour, whereas the Δ*ecfG2* deletion mutant did not produce this precipitate (Fig. 7). This is consistent with previous studies, showing that a mutant in *ecfG2* has a null ability to activate the GSR, including the expression of *nepR2* [50].

## Discussion

In this work, we describe an efficient genome editing procedure with potential applicability in sphingomonads, using * S. granuli* TFA as a model. Traditionally, mutational studies involving this group of bacteria have been developed via marked mutation or counterselection-mediated double recombination. The *sacB* gene has been the most frequently used counterselection marker, although alternative counterselection markers have been described, including *pheS*, which provides sensitivity to p-chlorophenylalanine [52]. However, there are representatives of this group that are naturally sensitive to this compound [24], making it not generally applicable to these procedures. Even in the case of *sacB*, previous reports have shown that its presence in the absence of selection can generate a certain toxicity that leads to accumulating mutations that inactivate it [53], thus rendering it ineffective for counterselection. This has also been observed when applying this strategy in members of the *Sphingomonadaceae* [24]. The optimised protocol we present here takes advantage of the natural streptomycin resistance of sphingomonads to indicate a successful first recombination, combined with a DNA double-strand break induction that efficiently triggers a second recombination. This strategy offers a fast-track procedure compared to the previously mentioned double recombination-based genome editing protocols.

Although we present an optimisation of the procedure for *S. granuli* TFA, the protocol is further amenable to different modifications that may tailor it to the specificities of other sphingomonads. For example, we use electrotransformation as a means to introduce plasmids in *S. granuli* TFA. However, both pMPO1412 (and the parental pEMG) and pSW-I are mobilisable via biparental or triparental mating, which may be an alternative for sphingomonads in which electrotransformation is not applicable. Furthermore, the selection markers in the pMPO1412 (and pEMG) and pSW-I can be exchanged according to the resistance profile of other sphingomonads. In this regard, several versions of pEMG and pSW-I derivatives with different selection markers are available through the Standard European Vector Architecture platform [54]. Another aspect that may be attuned to the requirements of other sphingomonads is the Sce-I expression induction step. In the case of *S. granuli*, the leaky expression of Sce-I, which is inducible by 3-methylbenzoate under the XylS-*Pm* system [41], was enough to trigger the second recombination. However, this expression system may behave differently in other species, and the addition of the inducer in the selection plates after introducing pSW-I may be required [42].

All in all, we describe a powerful tool for genome editing in *S. granuli* that can be further tailored to the requirements of other sphingomonad models.

## Summary step-by-step protocol

Here, we describe a step-wise protocol to perform this genome editing strategy on *S. granuli* TFA, which can be used as a base for optimisation to other sphingomonad species and laboratory methods (e.g. culture media, incubation times, selection/screening procedures). The protocol is described once the pMPO1412-derivative vector has been constructed and purified.

### First recombination event

Day 1: inoculation

- Inoculate 3 ml of MML broth with wild-type *S. granuli* TFA. Incubate at 30 °C, 180 r.p.m. to saturation (typically 24 h). The MML broth recipe is provided in Supplementary Materials and Methods.

Day 2: electrotransformation with the pMPO1412-derivative plasmid

- Prepare *S. granuli* TFA electrocompetent cells (our in-house protocol has been provided in the Supplementary Material). Alternatively, the pMPO1412 derivative can be introduced into the target strain by triparental mating.
- Electrotransform with 200 ng of the purified pMPO1412-derivative plasmid using an electroporator.
- Reconstitute the electroporated cell mixture with 1 ml of ice-chilled MML broth supplemented with 0.5 M sorbitol or 10 % glycerol. Incubate at 30 °C, 180 r.p.m. for 1.5 h.
- Perform serial dilution and plating on MML agar plates supplemented with kanamycin 20 mg l^−1^ for selection. Incubate the plates at 30 °C for 4–5 days.

Day 3: first recombination screening

- Perform dual streaking of 50–100 clones on 2 MML agar plates: 1 supplemented with kanamycin 20 mg l^−1^ and the other supplemented with kanamycin 20 mg l^−1^ and streptomycin 200 mg l^−1^. Incubate the plates at 30 °C for approximately 16 h.

Day 4: first recombination screening results

- By direct visualization, select the clones that grew well on MML agar with kanamycin only but grew more slowly on MML agar with kanamycin and streptomycin.
- To validate these recombinant clones, perform a colony PCR using primers specific to the plasmid introduced (we typically use primers annealing in the kanamycin resistance marker).
- Inoculate one of the validated cointegrate clones in 3 ml of MML broth to continue on to the second recombination event.

Day 5: electrotransformation with pSW-I

- Prepare electrocompetent cells of the cointegrate clone.
- Electrotransform the cointegrate clone with 200 ng of purified pSW-I.
- Reconstitute the electroporated cell mixture with 1 ml of ice-chilled MML broth supplemented with 0.5 M sorbitol or 10 % glycerol. Incubate at 30 °C, 180 r.p.m. for 1.5 h.
- Perform serial dilution and plating on MML agar plates supplemented with ampicillin 5 mg l^−1^ for selection. Incubate the plates at 30 °C for 4–5 days.

Day 6: second recombination screening

- Perform multiple streaking of 50 clones on 3 MML agar plates: 1 supplemented with kanamycin 20 mg l^−1^, one supplemented with ampicillin 5 mg l^−1^, and one supplemented with streptomycin 50 mg l^−1^. Incubate the plates at 30 °C for 24 h.

Day 7: second recombination screening results and mutation screening

- By direct visualization, select the clones that grew on MML agar with ampicillin only but did not grow on MML agar with kanamycin and ampicillin.
- To validate these recombinant clones and identify those that underwent the genetic modification, perform colony PCR using specific primers flanking the gene targeted for deletion in order to distinguish PCR products of different sizes between the wild-type and the mutant strain. Other modifications may require alternative validation approaches (e.g. other primer combinations for DNA insertions, Sanger sequencing for point mutation)
- Inoculate one of the validated mutant clones from those streaked on plain MML agar in 3 ml of MML broth. Start the curation on pSW-I. Incubate at 30 °C, 180 r.p.m. for 24 h.

Day 8: pSW-I curation

- Perform a passage of the mutant strain by performing a 1/500 dilution in fresh MML broth. Incubate at 30 °C, 180 r.p.m. for 24 h.

Day 9: pSW-I curation

- Repeat passaging as in day 8.

Day 10: pSW-I curation

- Streak on MML agar to obtain isolated colonies. Incubate at 30 °C for 24 h.

Day 11: validation of pSW-I-cured mutant clones

- Perform dual streaking of 50 clones on two MML agar plates: one supplemented with ampicillin 5 mg l^−1^ and a plain MML agar. Incubate the plates at 30 °C for 24 h.

Day 12: selection of pSW-I-cured clones

- By direct visualization, select the clones that grew on plain MML agar but did not grow on MML agar supplemented with ampicillin or kanamycin. Restreak on plain MML agar to obtain isolated colonies. Use these isolated colonies for cryoconservation of the newly generated mutant strain.

## supplementary material

10.1099/acmi.0.000755.v3 Uncited Supplementary Material 1.

## Abbreviations

Ap: ampicillin
ApR: ampicillin resistance/resistant
bp: base pair
GSR: general stress response
kb: kilobase pair
Km: kanamycin
KmR: kanamycin resistance/resistant
KmS: kanamycin sensitivity/sensitive
PCR: polymerase chain reaction
PEG: polyethylene glycol
PVA: polyvinyl alcohol
Str: streptomycin
StrR: streptomycin resistance
StrS: streptomycin sensitivity/sensitive
WT: wild type
X-gal: 5-bromo-4-chloro-3-indolyl-β-d-galactopyranoside
