# Supplementary material for: An improved genome editing system for Sphingomonadaceae
Source: Access Microbiol. 2024 May 13;6(5):000755.v3. doi: 10.1099/acmi.0.000755.v3 (PMC11165598; doi:10.1099/acmi.0.000755.v3)
Supplement: Uncited Supplementary Material 1. [file acmi-6-00755-s001.pdf]

An improved genome editing system for Sphingomonadaceae

Inmaculada García-Romero, Rubén de Dios and Francisca Reyes-Ramírez

Supplementary Material

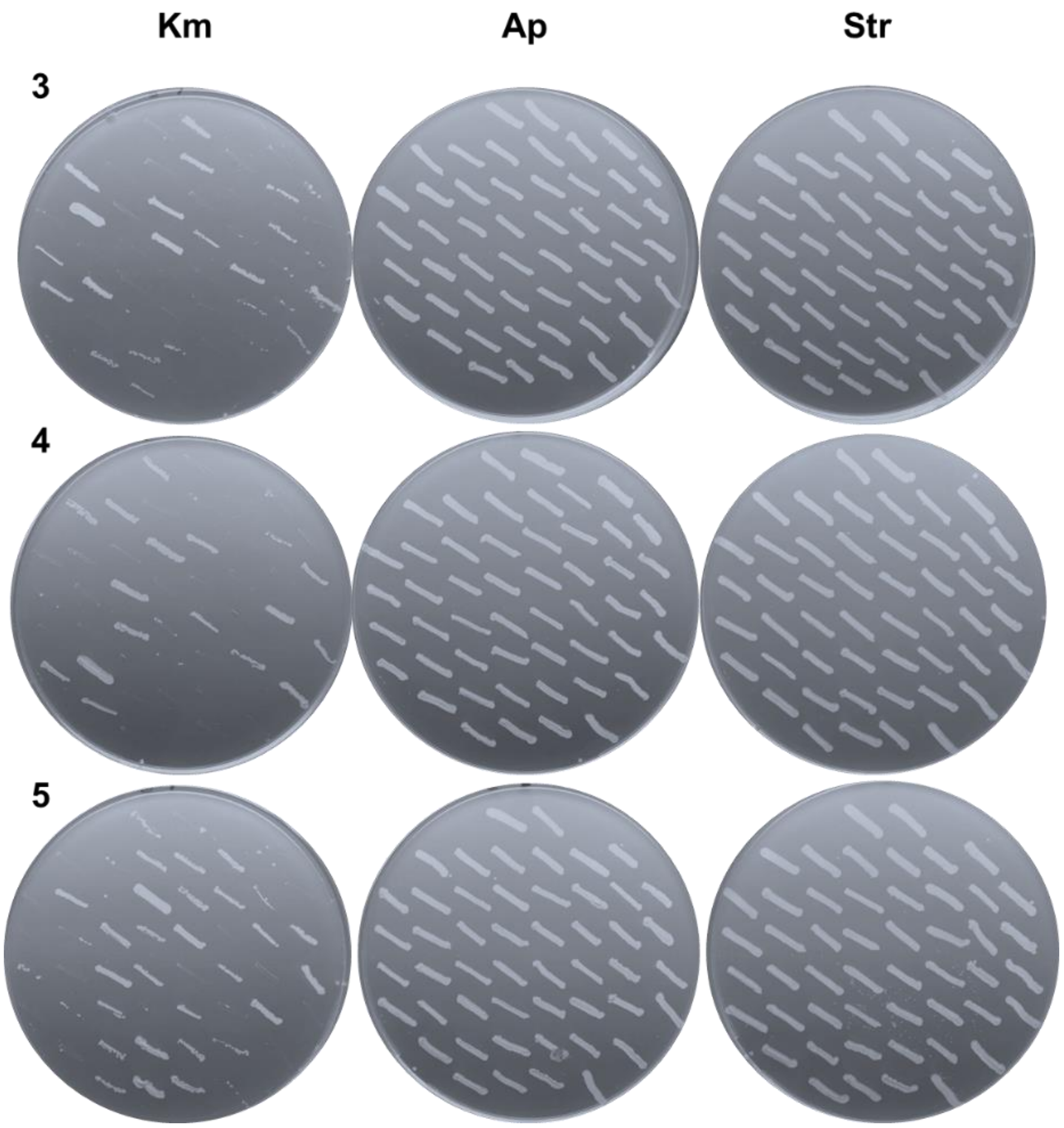

Supplementary Figure S1. Colony streaks of clones after electrotransformation with pSWI. The numbers 3, 4 and 5 indicates the clones of the first recombination event (see Figure 3) that were electrotransformed. Km, Ap and Str were used at 20 mg/L, 5 mg/L and 50 mg/L, respectively.

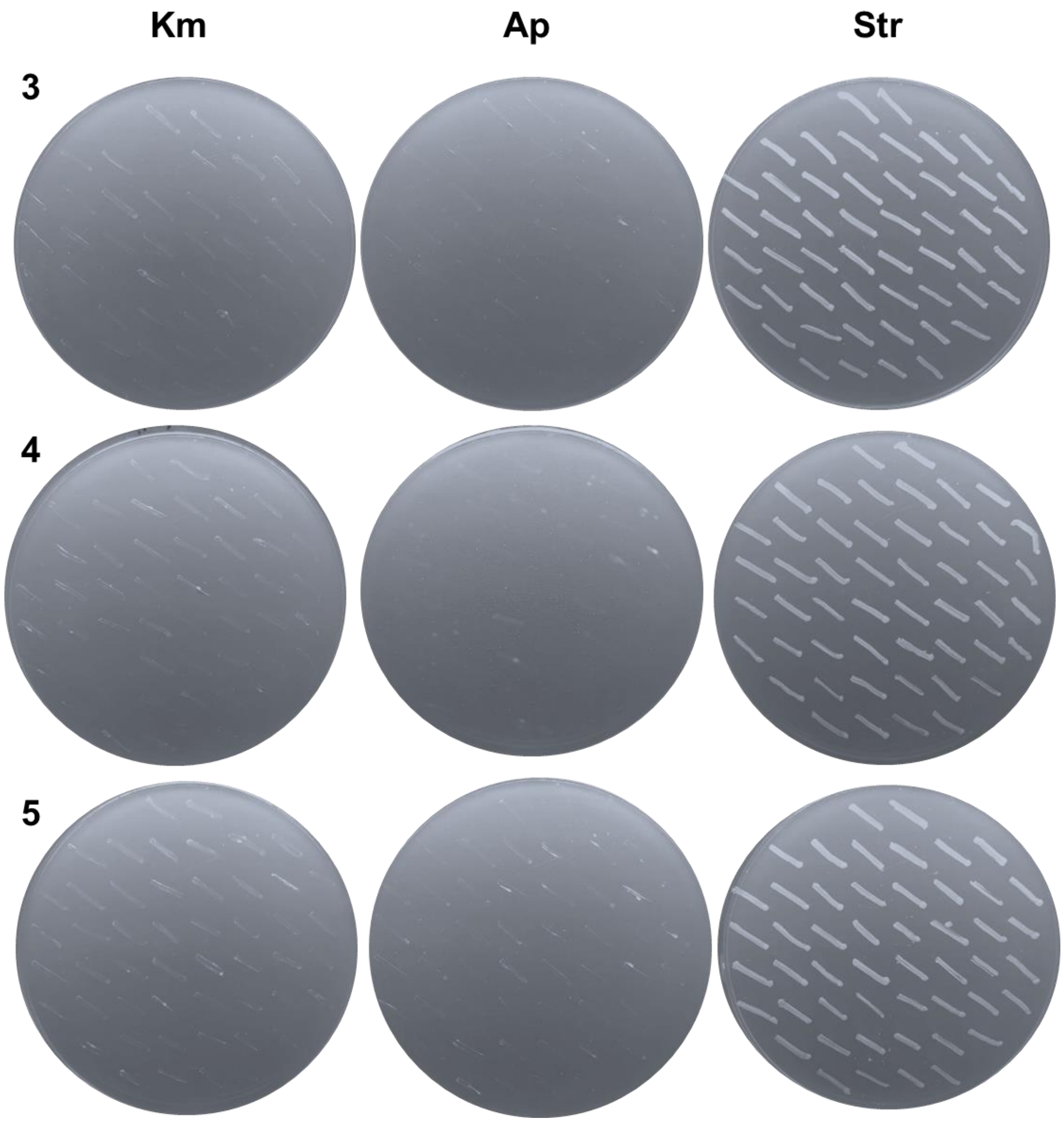

Supplementary Figure S2. Streaks of *ecfG2* mutant clones after curation of pSWI. The numbers 3, 4 and 5 indicates the clones obtained from the first recombination screening (see Figure 3) that were electrotransformed. Km, Ap and Str were used at 20 mg/L, 5 mg/L and 50 mg/L, respectively.

	Dilution 10^{-1} on Km (cfu)	Dilution 10^{-7} on Str (cfu)	Km^R frequency
**C- 1**	9	141	**6.38×10^{-8}**
**C- 2**	13	155	**8.39×10^{-8}**
**C- 3**	18	120	**1.50×10^{-7}**
**pMPO1409 1**	24	170	**1.41×10^{-7}**
**pMPO1409 2**	31	118	**2.63×10^{-7}**
**pMPO1409 3**	34	100	**3.40×10^{-7}**
**pMPO1162 1**	19	140	**1.36×10^{-7}**
**pMPO1162 2**	33	162	**2.04×10^{-7}**
**pMPO1162 3**	30	140	**2.14×10^{-7}**

Supplementary Table S1. Colony forming unit (cfu) counts and kanamycin resistance frequency (due to spontaneous emergence or plasmid recombination) after electrotransforming with plasmids pMPO1409, pMPO1162 or a water control and doing serial dilution and plating on MML agar supplemented with Km 20 mg/L for selection or with Str 50 mg/L for viable cell counting. Each replicate was performed with individually prepared *S. granuli* TFA electrocompetent cells.

	Dilution 10^{-1} on Ap (cfu)	Dilution 10^{-2} on Ap (cfu)	Dilution 10^{-8} on Str (cfu)	Ap^R frequency
**C-3**	0	0	99	0
**C-4**	9	0	71	1.27×10^{-8}
**C-5**	2	0	466	4.29×10^{-10}
**E3**	401	41	134	2.99×10^{-7}
**E4**	>1000	131	97	1.35×10^{-6}
**E5**	>1000	80	162	4.94×10^{-7}

Supplementary Table S2. Colony forming unit (cfu) counts and ampicillin resistance frequency (due to spontaneous emergence or plasmid acquisition) after electrotransforming with plasmid pSW-I or a water control and doing serial dilution and plating on MML agar supplemented with Ap 5 mg/L for selection or with Str 50 mg/L for viable cell counting. Each three replicates were performed with different cointegrate clones after receiving pMPO1162 (E3, E4, E5), numbered as in Figure 2.

Supplementary materials and methods

Culture media

Sphingopyxis granuli TFA strains were cultivated in rich MML liquid or solid medium [1] (2 g/L tryptone, 1 g/L yeast extract plus mineral medium [2] . When needed, the medium was supplemented with 20 mg/L kanamycin, 50 mg/L streptomycin, 200 mg/L streptomycin or 5 mg/L ampicillin.

Preparation of electrocompetent cells and electrotransformation

For the preparation of *S. granuli* TFA electrocompetent cells, 10 mL of MML were inoculated with a colony of the recipient strain and incubated at 30 °C for approximately 24 hours until saturation. This inoculum was subsequently diluted to an OD_{600} of 0.1 in 100 mL of fresh MML and incubated at 30 °C until an OD_{600} of 0.4 was reached. The culture was chilled on ice for 30 minutes, followed by cell collection through centrifugation at 5000 rpm for 10 minutes at 4 °C. The cells were then sequentially washed with 100 mL and 50 mL of ice-cold distilled water and 2 mL of ice-cold 10% glycerol (v/v). Finally, the cells were resuspended in 250 µL of 10% glycerol, and 40 µL aliquots were directly transformed or alternatively stored at -80 °C.

For electrotransformation, 200 ng of purified plasmid was mixed with an aliquot of electrocompetent cells. The mixture was transferred to a 2-mm wide electroporation cuvette and a 2.5kV current was applied with a Micropulser electroporator (BioRad). Cells were immediately reconstituted in 1 mL of MML supplemented with 0.5 M sorbitol of 10% glycerol and incubated 1.5 h at 30 °C, 180 rpm. Afterwards, the reconstituted cells were serially diluted, plated on selective media and incubated at 30 °C for 4-5 days.

Supplementary references

1. Hernáez, M.J., W. Reineke, and E. Santero, *Genetic analysis of biodegradation of tetralin by a Sphingomonas strain.* Appl Environ Microbiol, 1999. **65**(4): p. 1806-10.
2. Dorn, E., et al., *Isolation and characterization of a 3-chlorobenzoate degrading pseudomonad.* Arch Microbiol, 1974. **99**(1): p. 61-70.
